# [Retracted] Feroniellin A-induced autophagy causes apoptosis in multidrug-resistant human A549 lung cancer cells

**DOI:** 10.3892/ijo.2025.5736

**Published:** 2025-03-04

**Authors:** Chutima Kaewpiboon, Serm Surapinit, Waraporn Malilas, Jeong Moon, Preecha Phuwapraisirisan, Santi Tip-Pyang, Randal N. Johnston, Sang Seok Koh, Wanchai Assavalapsakul, Young-Hwa Chung

Int J Oncol 44: 1233-1242, 2014; DOI: 10.3892/ijo.2014.2297

Following the publication of this paper, it was drawn to the Editor's attention by a concerned reader that a pair of data panels (namely, the A549T-eto/FERO, 1 mM and FERO, 12 h panels) in the cell viability experiments shown in Fig. 1B on p. 1235 were partially overlapping, where the data had apparently been derived from the same original source. After having performed an independent review of the data in the Editorial Office, also in Fig. 1B, another pair of overlapping data panels were identified; moreover, strikingly similar data for the α-P-glycoprotein blots had been included in different figures (Figs. 1C and 3B), and there appeared to be a splicing event/break in the continuity of the α-actin bands shown for the control data in Figs. 1C and 3B that wasn't apparent elsewhere for the proteins of interest shown in these figure parts.

Owing to these possible anomalies and the duplications of the data that have been identified in this paper, the Editor of *International Journal of Oncology* has decided that it should be retracted from the Journal on account of a lack of confidence in the presented data. After having been in contact with the authors, they accepted the decision to retract the paper. The Editor apologizes to the readership for any inconvenience caused.

